# Spontaneous preterm birth: the underpinnings in the maternal and fetal genomes

**DOI:** 10.1038/s41525-021-00209-5

**Published:** 2021-06-08

**Authors:** Esha Bhattacharjee, Arindam Maitra

**Affiliations:** National Institute of Biomedical Genomics, Kalyani, India

**Keywords:** Medical genomics, Genetics research

## Abstract

Preterm birth (PTB) is a major cause of neonatal mortality and health complications in infants. Elucidation of its genetic underpinnings can lead to improved understanding of the biological mechanisms and boost the development of methods to predict PTB. Although recent genome-based studies of both mother and fetus have identified several genetic loci which might be implicated in PTB, these results suffer from a lack of consistency across multiple studies and populations. Moreover, results of functional validation of most of these findings are unavailable. Since medically indicated preterm deliveries have well-known heterogeneous causes, we have reviewed only those studies which investigated spontaneous preterm birth (sPTB) and have attempted to suggest probable biological mechanisms by which the implicated genetic factors might result in sPTB. We expect our review to provide a panoramic view of the genetics of sPTB.

## Introduction

Preterm birth (PTB) is defined as any live birth before 37 completed weeks of gestation and is the major cause of mortality in neonates and infants^1^. Globally, about 15 million preterm babies are born per year, among which around 3.5 million (23.4%) PTBs take place in India alone^2^. Neonates born early preterm (<34 weeks) have higher mortality and morbidity risks while the ones born late preterm (between 34 and 37 weeks) are at increased risk of health complications, such as, respiratory disorders, cardiovascular disorders, infections, feeding difficulties, visual and hearing problems and learning disabilities as compared to early term (37 to <39 weeks) and full-term (39–40 weeks) neonates^2–4^, which are likely due to incomplete development of fetal organs and systems. They are also at increased risk of late-onset disorders, such as obesity, metabolic syndrome, hypertension, and type 2 diabetes in early life^5^.

**Fig. 1:**
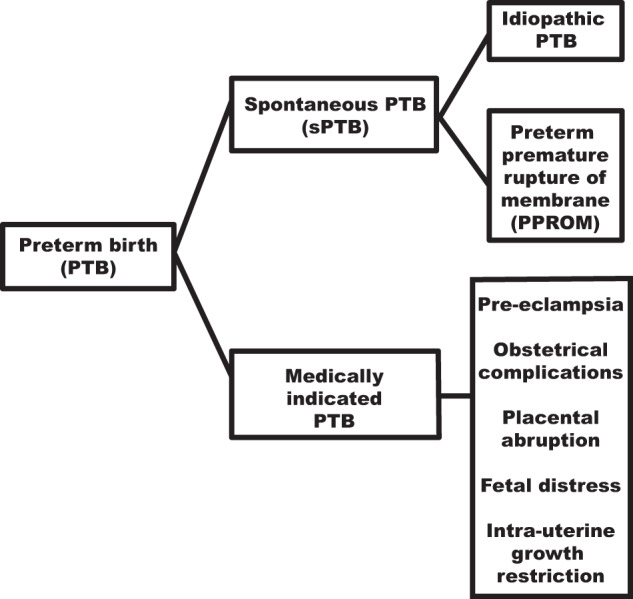
Schematic representation of the categories of preterm birth. Spontaneous PTB and medically indicated PTB are two subtypes of preterm birth (PTB). Spontaneous PTB can be further subdivided into idiopathic PTB and preterm premature rupture of membrane (PPROM). In idiopathic PTB, uterine contractions and cervical dilation is followed by rupture of the fetal membrane before the expulsion of the baby out of the womb while in PPROM, the fetal membranes get ruptured before the initiation of uterine contractions before 37 weeks of gestational age. Medically indicated PTB can be further classified into pre-eclampsia, obstetrical complications, placental abruption, fetal distress, and intrauterine growth restriction.

Clinically, PTB is classified into several subtypes (Fig. 1). About 40–45% of preterm deliveries are idiopathic in nature with spontaneous onset of labor; 25–30% involve preterm premature rupture of membrane (PPROM) while 30–35% are medically indicated^6^.

**Fig. 2:**
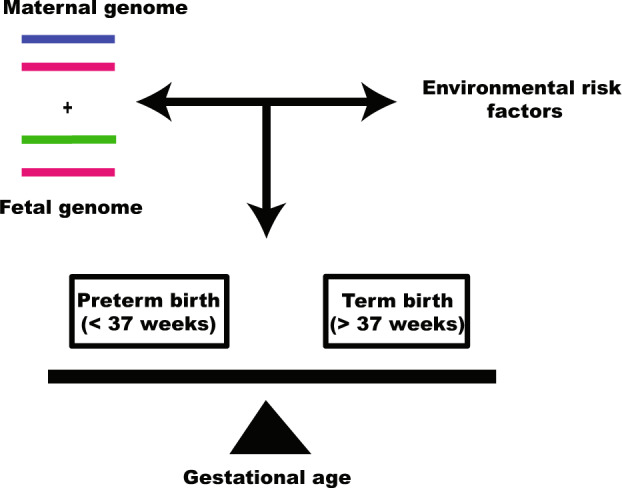
Major risk factors that influence the birth outcome. Interactions between maternal and fetal genome with environmental risk factors determine the period of gestation at delivery. Genome with high-risk alleles in the background of environmental risk factors leads to preterm birth.

Being a multifactorial trait, both environmental and genetic risk factors contribute to the pathophysiology of PTB (Fig. 2). Here, we provide a review of results from the genome-based studies of spontaneous PTB, with a focus on the biological relevance of those genetic factors that have been either replicated or functionally validated. Since the etiology of medically-indicated PTB is heterogeneous as its determinants include pre-eclampsia, fetal distress, intrauterine growth retardation, etc.^7^, we have restricted our discussions to sPTB only.

## Genetic underpinnings of PTB

In addition to the risk factors of PTB identified in various epidemiological studies (Supplementary Table 1), family-based^8,9^, twin-based^10,11^ and intergenerational studies^12,13^ have provided evidence of genomic factors underlying PTB. Estimates of heritability of PTB identified from twin studies range between 17% and 36%^10,11^. PTB is a clinical entity that is defined by the genomes of two individuals—the mother and the fetus. Findings of multiple epidemiological studies have suggested increased maternal genetic contribution to PTB either through the mother’s genome or maternally inherited variants in the fetal genome compared to those inherited paternally^8,12,14^. Another study reported that between the parents, mothers born preterm had a higher risk of delivering the first child at preterm (relative risk 1.54; 95% confidence interval (CI): 1.42, 1.67) than the preterm-born fathers (relative risk 1.12; 95% CI: 1.01, 1.25)^15^.

## Genomic approaches to study sPTB

Three main approaches have been used to identify genetic factors that contribute to the risk for sPTB which are discussed below.

### Genome-wide linkage studies

Initially, genome-wide studies on mothers and infants were conducted using linkage analysis to identify genetic factors contributing to spontaneous PTB. However, none of these could identify any maternal genomic locus significantly linked to sPTB. Genome-wide linkage analysis of infants using autosomal markers in seven clinically characterized sPTB multiplex families from Northern Finland identified three intronic markers rs2684811, rs1521480, rs4966936 (heterogeneity LOD or HLOD > 3) within insulin-like growth factor 1 receptor (*IGF1R*) gene on 15q26.3 locus to be linked to sPTB^16^. A haplotype was also identified in this gene which showed co-segregation with sPTB in six out of the seven families and this was further validated in the Lund–Malamö dataset^16^. However, a subsequent association study based on SNPs in this candidate gene in the Finnish population (334 cases and 197 controls) could not identify any SNP associated with sPTB after permutation correction^16^. This might be due to the small sample size used. Although the investigators concluded that ligands of IGF1R that regulate IGF1R dependent signaling cascades are associated with sPTB^16^, how dysregulation of IGF1R signaling itself can be involved in sPTB was not addressed. A candidate gene-based association study on the Chinese population identified an SNP in *IGF1R* (rs2229765) which is associated with reduced risk of sPTB in women with GA or AA genotype as compared to the women with GG genotype^17^. A study in mouse and human breast tumor epithelial cell lines reported that downregulation of *IGF1R* expression increases cellular stress and cytokine (IL-6 and CCL2) production^18^. Since oxidative stress^19^ and proinflammatory cytokine-mediated inflammatory pathways^20^ have been previously reported to be involved in sPTB, hence downregulation of *IGF1R* expression might lead to sPTB through either or both of these pathways.

A significant linkage signal was identified on Xq13.1 (rs6525299; HLOD 3.72) for sPTB^21^ in the same set of families that were included in the study previously mentioned^16^. Case-control association studies on preterm and term delivering mothers as well as preterm and term infants conducted in the Finnish population with the candidate genes located near the linkage signal identified the expansion of CAG repeats (≥26) in exon 1 of androgen receptor (*AR*) gene in Xq12 to be over-represented among the preterm infants (*P* = 0.0006)^21^. Interestingly, it had been suggested earlier that molecular crosstalk between *IGF1R* and *AR* might lead to sPTB^21^. Later studies have shown that a longer polyglutamine chain in the N-terminal transactivation domain of AR hinders transactivation activity^22^ resulting in decreased AR signaling which leads to apoptosis via activation of Caspase-3^23^. Since apoptosis in the placenta is involved in sPTB^24^, decreased AR signaling might lead to sPTB via apoptosis in the placenta.

Genome-wide linkage analysis using microsatellite markers in 288 Mexican-American families found 18q21.33-q23 locus (LOD = 3.6) to be significantly linked to sPTB. The plasminogen activator inhibitor type 2 (*PAI-2*) gene was suggested as an important candidate gene in this region^25^ as this gene was found to be significantly associated with sPTB in the Australian population earlier^26^. Subsequent studies in mice models revealed that *Pai-2* plays an important role in inducing autophagy by enhancing the expression of Beclin1 which induces autophagosome formation^27,28^. Autophagy being associated with reduced PTB^29^, alteration of *PAI-2* expression might modulate sPTB risk by effecting change in autophagy.

### Genome-wide association studies (GWAS)

**Fig. 3:**
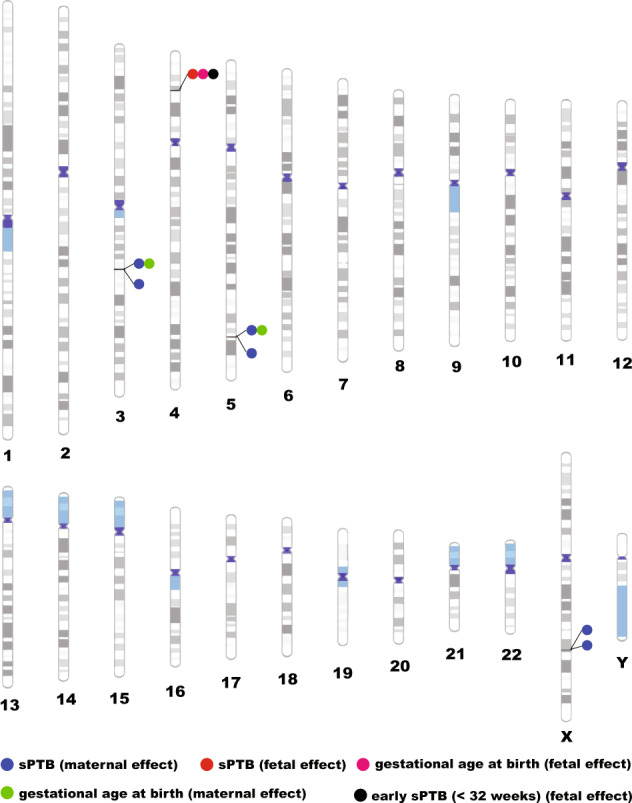
Results of the genome-wide association studies. Only those loci which were associated with sPTB at minimally suggestive significance (*P* < 1 × 10^−6^) in discovery stage and either got replicated or functionally validated are shown in this figure. Blue circles are showing maternal genetic associations with sPTB while a red circle is showing fetal genetic association with sPTB. The pink circle is showing fetal genetic variant which was also reported to be associated with gestational age at birth while green circles are showing maternal genetic variants which were also reported to be associated with gestational age at birth. The black circle is showing the association of the maternal genetic variant with early sPTB (less than 32 weeks).

Although more than 100 candidate genes were found to be associated with sPTB in candidate gene-based studies, most of the findings suffer from a lack of replication^30^. The probable reason for this might be the heterogeneity of underlying genetic causes of sPTB, population-specific risk factors, and limited sample size. In contrast to these, some recent studies have used genome-wide association analysis to identify common variants associated with sPTB. In order to guard against discussing false-positive results in this review, we have included only those association studies in which findings have attained at least suggestive significance level (*P* < 1 × 10^−6^) and were either replicated or functionally validated (Fig. 3).

A two-stage genome-wide association study, which used data from 43,568 women of European ancestry of whom 3331 women had spontaneous preterm deliveries, found the maternal loci in *EBF1* (rs2963463 and rs2946169) and *EEFSEC* (rs201450565 and rs200745338) genes, to be significantly associated (*P* < 5 × 10^−8^) with sPTB. The results were replicated in 8643 women in three Nordic datasets among which 2565 women delivered preterm. Association of variants in *AGTR2* (rs201386833 and rs5950506) with sPTB achieved genome-wide significance (*P* < 5 × 10^−8^) in a joint analysis of discovery and replication sets. Along with loci in these three genes, the association of *WNT4* variants with gestational duration also achieved genome-wide significance level both in the discovery and replication datasets^31^. The investigators concluded from the functional studies that the implicated variant in *WNT4* results in longer gestational duration, suggestive of protecting women from delivering preterm^31^. However, functional analyses of none of the variants associated with sPTB were performed. The biological relevance of the genes found associated with sPTB is discussed below.

#### EBF1

Early B-cell factor 1 encoded by *EBF1*, is essential for normal B-cell development^31^. Low EBF1 mRNA in the second and/or third trimester is significantly associated with an elevated risk of PTB which might be due to the involvement of maternal-fetal immune pathway and/or apoptosis^32^ of the fetal membranes leading to their early rupture.

#### EEFSEC

*EEFSEC* encodes eukaryotic elongation factor selenocysteine tRNA-specific protein, which incorporates essential micronutrient selenocysteine, into selenoproteins. Selenoproteins maintain cellular redox status by antioxidant defenses and also modulates inflammatory responses^31^. Moreover, it plays an important role in reproduction^33^. Earlier studies have linked these physiological processes to PTB^31^. Alteration in *EEFSEC* expression may lead to sPTB via any of these pathways.

#### AGTR2

*AGTR2* encodes angiotensin II receptor type 2 which modulates uteroplacental circulation^31^. rs7889204 is an intergenic variant located near *AGTR2*. Decreased binding of CEBPB (CCAAT Enhancer Binding Protein Beta) and HOXA10, a homeobox protein, to rs7889204-C allele results in decreased *AGTR2* expression which might lead to sPTB^34^. Since higher uterine and umbilical artery resistance indices across gestation were found to be associated with sPTB^35^, decreased *AGTR2* expression might result in compromised uteroplacental circulation ultimately leading to sPTB.

A GWAS conducted on 247 infants with spontaneous PTB (less than 36 weeks) and 419 term controls (38–41 weeks) of Finnish descent identified rs116461311 in Slit homolog 2 (*SLIT2*) gene to be strongly associated with sPTB (*P* = 1.6 × 10^−6^). It was also overrepresented in 172 very preterm born infants (less than 32 weeks) (*P* = 1.55 × 10^−7^). This SNP also exhibited a highly significant association with gestational age at delivery (*P* = 3.1 × 10^−7^). Silencing of *SLIT2* and its receptor roundabout homolog 1 (*ROBO1*) gene in trophoblasts resulted in the downregulation of proinflammatory cytokines^36^. As upregulation of proinflammatory cytokines stimulates uterine myometrial contractions, it was proposed that upregulation of *SLIT2* and *ROBO1* might result in premature activation of uterine myometrium and sPTB^36^.

### Rare variant studies

Most studies to date have focused on identifying common variants from both maternal and fetal genomes, associated with sPTB. But individually the common variants have been found to confer only small increments in sPTB risk. The hunt for rare variants with large effects has been launched in sPTB only recently, based on multiplex families with more than one woman in each family delivering preterm.

Whole exome sequencing (WES) of two mother-daughter pairs from Finnish multiplex families with sPTB identified novel missense variants in genes belonging to the complement cascade pathway. This pathway was also found to be associated in exome analysis of six additional mothers delivering preterm babies. Genes from this pathway were investigated for association analysis in Finnish nuclear families (237 cases and 328 controls), resulting in the identification of three SNPs in *CR1*, the strongest association being with an exonic SNP, rs6691117 (*P* = 1.07 × 10^−4^, OR = 1.73 (95% CI: 1.31–2.29)) which even surpassed Bonferroni corrected *p* value^37^. *CR1* encodes complement C3b/C4b Receptor 1 which is located on the surface of the erythrocytes. Although normally erythrocyte sedimentation rate (ESR) increases in pregnancy to clear immune complexes from the walls of blood vessels, this SNP is associated with decreased ESR indicating that the risk promoting allele might lead to greater systemic inflammation due to non-clearance of immune complexes^37^.

A recent WES on seventeen mothers from seven Finnish multiplex families with recurrent sPTB, identified rare variants in the genes in the glucocorticoid receptor (GR) signaling pathway (*P* < 1.7 × 10^−8^). This pathway was replicated in ninety-three Danish sister pairs. Missense variants in *HSPA1L* had a population-specific and family-specific effect in the manifestation of sPTB, which is generally a characteristic feature of rare variants with large effect sizes. A non-synonymous variant rs34620296 (Ala268Thr), identified in four Finnish families, generates an additional phosphorylation site near the nucleotide-binding site of HSPA1L (a member of the HSP70 family) affecting its interaction with ADP or its own stability^38^, reducing its chaperone activity^39^. The Hsp70 and Hsp90 chaperones as well as their co-chaperones form a heteromeric complex. The binding of this complex to the GR is essential for the maturation of GR^40^. GR signaling pathway cross-talks with estrogen signaling^41^ and progesterone signaling^42^ pathways for efficient regulation of the pro- and anti-inflammatory microenvironment in reproductive tissues during pregnancy. Impairments in the GR signaling pathway might result in a shift from anti-inflammatory to the pro-inflammatory microenvironment, causing premature activation of labor^43^. Interestingly, a nonsynonymous *HSPA1L* SNP, rs2075800, was previously found to be associated with sPTB in African-American women^44^. Furthermore, a meta-analysis using pathway analysis indicated an association of *HSPA1L* with sPTB^45^. This indicates an important role of this gene in spontaneous PTB via the glucocorticoid signaling pathway.

### Integrative “omics” approaches

With the advancement in “omics” technologies, significant progress has been achieved in the identification of biological correlates of complex phenotypes. Since individually each of these technologies is unlikely to capture the entire biological complexity of complex phenotypes, integration of the findings from multi-omics approaches is recommended. Integration of whole-genome sequencing, RNA-sequencing, and DNA methylation results from more than 600 families of mixed ancestry resulted in the identification of association of *RAB31* and *RBPJ* with very early PTB (<28 weeks) in European and American population respectively even after adjusting for population stratification^46^. Possible mechanisms by which alterations of these genes lead to PTB are as follows:

#### RAB31

RAB31, a member of Ras GTPases, plays a key role in the internalization of ligand-bound EGFR (epidermal growth factor receptor) from the cell membrane into endosomes through receptor-mediated endocytosis^47^. EGFR signaling plays an important role in cellular proliferation, differentiation, migration, and also angiogenesis^48^. Since disruption of placental angiogenesis leads to PTB^49^, altered *RAB31* expression may lead to altered EGFR signaling resulting in PTB via disrupted placental angiogenesis.

#### RBPJ

RBPJ (recombination signal binding protein for Immunoglobulin kappa J region) is an important transcriptional regulator of the Notch signaling pathway. Premature activation of Notch signaling might increase the inflammatory response upon the enhancement of NF-ĸβ (nuclear factor kappa beta) activity^50^, ultimately leading to preterm labor^51^.

## Do mitochondrial genomic variants predispose to sPTB?

Since the mitochondrial genome is maternally inherited, the enhanced maternal genetic contribution to PTB suggests an association of mitochondrial DNA variants with PTB^16^. Although a case-control study of mothers and infants belonging to Danish and Norwegian populations failed to detect any association between polymorphisms in mitochondrial genome and PTB^52^, a retrospective cohort study on Dutch women found that those harboring the mitochondrial mutation mt.3243A>G in *MT-TL1* have a higher risk of PTB (25.3%) as compared to the general Dutch population (7.4%). Out of 25.3% of women who delivered preterm, about half of them experienced spontaneous preterm delivery^53^. *MT-TL1* encodes for a tRNA (Leu-UUR) which plays a critical role in the translation of proteins essential for the assembly and function of the mitochondrial complexes in the oxidative phosphorylation pathway^54^. Increased ROS production due to dysregulation of respiratory chain complexes might lead to placental dysfunction^55,56^ resulting in adverse birth outcomes^57,58^.

Another study found a greater degree of divergence of mitochondrial ancestry in infants born preterm as compared to those born at term (705 cases and 721 controls; *P* = 0.046) in the GPN dataset. This finding was replicated in BPD/HRS dataset where 1724 cases were from the BPD dataset and 12,507 controls were from the HRS dataset^59^. However, it could not be replicated in the GENEVA dataset (1035 cases and 508 controls)^59^. Since mtDNA and nuclear genomic polymorphisms that are involved in the formation of multi-subunit complexes of electron transport chain have co-evolved, mitochondrial haplogroup containing markers of one ancestry in the background of a nuclear genome with different ancestral origin might result in impaired mitochondrial potential which may partially explain the elevated risk of PTB in African-American population^59^.

## Gene–environment interactions in sPTB

Most of the genetic studies on sPTB have not taken into account the gene–environment interactions. Examining only the direct associations of traits with variants may result in missing those which influence sPTB in conjunction with specific environmental exposures. In sPTB and other pregnancy-related complexities, two different environmental components are involved - in utero environment to which the fetus is exposed and the external environment to which the mother is exposed. Understanding maternal gene–environment interactions might help to detect high-risk sub-groups in the population while understanding fetal gene–environment interactions might provide insights into the biology behind the pregnancy-related complexities.

Analysis of genome-wide gene–environment interactions identified maternal *COL24A1* variants having significant interaction with maternal pre-pregnancy BMI on the risk of sPTB with rs11161721 as the most significantly associated variant (*P* = 1.8 × 10^−8^) in subcutaneous adipose tissue. This study was conducted on 1733 African-American women (698 cases and 1035 controls) from the Boston Birth Cohort^60^. It is the first study that not only explored genome-wide gene–environment interactions in PTB by offering insights into the “missing heritability” of sPTB but was also able to replicate the findings in the same ethnic population from the GPN dataset deposited in dbGaP^60^. However, this finding could not be replicated in the Caucasian population^60^ which might suggest a population-specific role of this SNP in sPTB. Women with normal body weight and harboring rs11161721-AA genotype and obese women with rs11161721-CC genotype were found to be at elevated risk for sPTB. rs11161721-A allele was reported to be associated with higher *COL24A1* expression than rs11161721-C allele in subcutaneous adipose tissue (*P* = 8.2 × 10^−8^). *COL24A1* encoded collagen type XXIV alpha 1 protein regulates type I collagen fibrillogenesis during fetal development. Type I collagen being a component of the extracellular matrix (ECM), influences the functions of many cells and organs. Altered *COL24A1* expression may impact the proper functioning of ECM resulting in pathological events which might lead to sPTB^60^.

## Discussion

sPTB being a multifactorial trait involving both the mother and the fetus, genetic studies of sPTB should consider contributions of both maternal and fetal genomes, as well as gene–environment interactions since individual’s genotypes interacting with both the external and in utero environmental factors, may have the potential to modify the risk. Although multiple genomic variants have been reported to be associated with sPTB, many of these associations could not be replicated^61–63^ and the results were found to be inconsistent across different studies conducted even in the same population. In some cases, the association did not reach genome-wide significance^64,65^ possibly because of a lack of statistical power. In addition, most of the studies which have analyzed genomics of sPTB as a dichotomous trait have excluded the borderline gestational ages to avoid misclassification. This might have led to inconsistency in defining preterm and term births across these studies.

Functional validation of these discoveries is essential for a deep insight into the role of the implicated variants in sPTB since the biological effects of the variants may not be always mediated through the nearest genes. The human placenta is a unique organ due to the presence of numerous human-specific molecular features^66^. As a result, the utility of extrapolation of the findings in animal models to the human system is uncertain. Alternatively, appropriate cell lines of human origin might provide a better choice. For evaluation of the effect of genotypes, specific analytes can be measured in maternal peripheral blood or umbilical cord blood and amniotic fluid as well.

Although PTB is a global phenomenon, most studies have been conducted on families and individuals belonging only to populations of European and African-American ancestries. These studies can be expanded to other populations, emphasizing those who have a substantial prevalence of sPTB. Recently, for improved prediction of a person’s genetic susceptibility to common chronic diseases, polygenic risk scores (PRS) are being calculated using genome-wide genetic data which can be applied to sPTB as well. But the main concern with PRS is that it cannot yet predict effectively in non-European populations. GARBH-Ini, a cohort of pregnant women has been established in India to provide insights into the biological correlates of PTB using an integrative-omics approach^67^. Such integrative-omics approaches might help to identify the biological mechanisms through which inter-individual variation results in sPTB.

## Conclusion

Early detection of the risk of PTB is invaluable to combat this global health burden of adverse pregnancy outcome. As compared to biochemical markers, genomic markers can identify high-risk women even prior to pregnancy. The studies discussed by us were those where findings were either reproducible in a different set of participants or were validated functionally. Our selection criteria, while ensuring that we reviewed only those results which are unlikely to be false positive, might have also resulted in exclusion of findings that are of biological relevance without either attaining statistical significance or which could not be replicated or biologically validated. With improved phenotyping of sPTB viz. premature onset of labor, PPROM, cervical insufficiency and further functional validation of genetic associations, the findings of the studies as those included in the review are expected to contribute towards better prediction of sPTB and development of targeted clinical interventions to reduce the same.

## Supplementary information

Supplementary Information

## Data Availability

Since this is a review, all information used for the manuscript are from research publications which have been appropriately cited in the manuscript.
