# Supplementary material for: Spontaneous preterm birth: the underpinnings in the maternal and fetal genomes
Source: NPJ Genom Med. 2021 Jun 8;6:43. doi: 10.1038/s41525-021-00209-5 (PMC8187433; doi:10.1038/s41525-021-00209-5)
Supplement: Supplementary file 1 — Supplementary Information [file 41525_2021_209_MOESM1_ESM.pdf]

Supplementary Table 1

Factors associated with preterm birth. Strength of the associations was not considered but these may assist in better controlling genetic and epigenetic studies in the future.

Risk factor	Explanation	Reference
Maternal age at pregnancy	Both younger and aged pregnant women are at increased risk for PTB as compared to the women of 30-34 years age	Fuchs F, *et al,* 2018
Multiple pregnancy	Multiple pregnancy increases PTB risk as compared to singletons	Kurdi AM, *et al*, 2004
Parity	Lower the parity, more the risk of PTB	Mayo JA, *et al*, 2017; Delnord M, *et al*, 2018
Interpregnancy interval	Shorter interpregnancy interval increases PTB risk	Smith GCS, *et al*, 2003
Infant sex	Pregnant women carrying male fetus have an increased risk for PTB	Peelen MJCS, *et al*, 2016
Lifestyle	Women who smoke during pregnancy are at increased risk for PTB<br>Alcohol consumption during pregnancy increases PTB risk	Ion R, *et al*, 2015<br>Albertsen K, *et al*, 2004
Nutrition	Lower serum folic acid level in pregnant women increases PTB risk;<br>Vitamin D deficiency increases PTB risk while its supplementation during pregnancy reduces the risk	Liu X, et al, 2016<br>Zhou SS, et al, 2017
Mental health	Maternal psychological and social stress increases the risk for PTB<br>Antenatal depression is associated with PTB	Lilliecreutz C, *et al*, 2016<br>Mochache K, *et al*, 2018
Maternal pre-pregnancy BMI	Both underweight and obese mothers are at increased risk for PTB	Han Z, *et al*, 2011; Oken E, *et al*, 2019
Ethnicity	African-American women have increased risk for PTB	Whitehead N, *et al*, 2016

References (mentioned in this file):

1. Fuchs, F., Monet, B., Ducruet, T., Chaillet, N., Audibert, F. Effect of maternal age on the risk of preterm birth: A large cohort study. *PLoS One*. **13**, e0191002 (2018).
2. Kurdi, A. M., Mesleh, R. A., Al-Hakeem, M. M., Khashoggi, T. Y., Khalifa, H. M. Multiple pregnancy and preterm labor. *Saudi Med J*. **25**, 632–637 (2004).
3. Mayo, J. A., Shachar, B. Z., Stevenson, D. K., Shaw, G. M. Nulliparous teenagers and preterm birth in California. *J Perinat Med*. **45**, 959–967 (2017).
4. Delnord, M., Blondel, B., Prunet, C., Zeitlin, J. Are risk factors for preterm and early-term live singleton birth the same? A population-based study in France. *BMJ Open*. **8**, e018745 (2018).
5. Smith, G. C. S., Pell, J. P., Dobbie, R. Interpregnancy interval and risk of preterm birth and neonatal death: retrospective cohort study. *BMJ*. **327**, 313 (2003).
6. Peelen, M. J. C. S., *et al.* Impact of fetal gender on the risk of preterm birth, a national cohort study. *Acta Obstet Gynecol Scand*. **95**, 1034–1041 (2016).
7. Ion, R. and Bernal, A. L. Smoking and preterm birth. *Reprod Sci*. **22**, 918–926 (2015).
8. Albertsen, K., Andersen, A. M., Olsen, J., Grønbaek, M. Alcohol consumption during pregnancy and the risk of preterm delivery. *Am J Epidemiol*. **159**, 155–161 (2004).
9. Liu, X., *et al.* Folic acid supplementation, dietary folate intake and risk of preterm birth in China. *Eur J Nutr*. 55, 1411–1422 (2016).
10. Zhou, S. S., Tao, Y. H., Huang, K., Zhu, B. B., Tao, F. B. Vitamin D and risk of preterm birth: Up-to-date meta-analysis of randomized controlled trials and observational studies. *J Obstet Gynaecol Res*. **43**, 247–256 (2017).
11. Lilliecreutz, C., Larén, J., Sydsjö, G., Josefsson, A. Effect of maternal stress during pregnancy on the risk for preterm birth. *BMC Pregnancy Childbirth*. **16**, 5 (2016).

12. Mochache, K., Mathai, M., Gachuno, O., Vander Stoep, A., Kumar, M. Depression during pregnancy and preterm delivery: a prospective cohort study among women attending antenatal clinic at Pumwani Maternity Hospital. *Ann Gen Psychiatry*. **17**, 31 (2018).

13. Han, Z., Mulla, S., Beyene, J., Liao, G., McDonald, S. D. Maternal underweight and the risk of preterm birth and low birth weight: a systematic review and meta-analyses. *Int J Epidemiol*. **40**, 65–101, (2011).

14. Oken, E., Aris, I. M., Young, J. G. Pre-pregnancy weight and preterm birth: a causal relation? *Lancet Diabetes Endocrinol.* **7**, 663–665 (2019).

15. Whitehead, N. and Helms, K. Racial and ethnic differences in preterm delivery among low-risk women. *Ethn Dis.* **20**, 261–266 (2010).
